# Double-Kissing Nanocrush for Bifurcation Lesions: Development, Bioengineering, Fluid Dynamics, and Initial Clinical Testing

**DOI:** 10.1016/j.cjca.2019.08.037

**Published:** 2020-06

**Authors:** Paul D. Morris, Rebecca Gosling, Alex Rothman, Javaid Iqbal, Claudio Chiastra, Monika Colombo, Francesco Migliavacca, Amerjeet Banning, Julian P. Gunn

**Affiliations:** a Department of Infection, Immunity and Cardiovascular Disease, University of Sheffield, Sheffield, United Kingdom; b Sheffield Teaching Hospitals, NHS Foundation Trust, Sheffield, United Kingdom; c Insigneo Institute for *In Silico* Medicine, University of Sheffield, Sheffield, United Kingdom; d Laboratory of Biological Structure Mechanics, Department of Chemistry, Materials and Chemical Engineering “Giulio Natta,” Politecnico di Milano, Milan, Italy; e Materials in Bionanotechnology and Biomedical Laboratory, Department of Mechanical and Aerospace Engineering, Politecnico di Torino, Turin, Italy; f Department of Cardiology, Glenfield Hospital, University Hospitals of Leicester, NHS Trust, Leicester, United Kingdom; g Department of Cardiovascular Sciences, University of Leicester, Leicester, United Kingdom

## Abstract

**Background:**

When possible, a single-stent technique to treat coronary bifurcation disease is preferable. However, when 2 stents are required, there is scope to improve on existing techniques. The crush technique has already been improved with the introduction of double-kissing (DK) and minicrush. We sought to refine and simplify the minicrush technique, retaining its advantages while avoiding its disadvantages, by developing a DK nanocrush technique.

**Methods:**

The DK nanocrush method allows complete lesion coverage of a bifurcation lesion without excessive metal layers. This is achieved by positioning the side branch (SB) stent with minimal protrusion into the main branch (MB), implantation of the SB stent with an undeployed balloon in the MB, immediate kissing-balloon inflation with formation of a minimal neocarina, stenting the MB, recrossing the proximal part of the SB without crossing a double metal layer, and final kissing. We demonstrate this technique with benchtop implantation, microscopic computed tomographic reconstruction, computational fluid dynamics (CFD) modelling, and clinically with the use of angiographic and intravascular imaging.

**Results:**

The DK nanocrush was practically feasible and resulted in full ostial coverage. CFD analysis demonstrated minimally disturbed blood flow. The technique was successfully utilised in 9 patients with bifurcation lesions with excellent angiographic outcomes and no adverse events over 12 months.

**Conclusions:**

The DK nanocrush technique may represent the ultimate refinement of the original crush technique with a number of practical and theoretical advantages. It remains to be tested against other bifurcation techniques in prospective trials.

## Résumé

**Introduction:**

Lorsque possible, la technique de l’endoprothèse unique est préférable pour traiter des lésions de la bifurcation coronaire. Toutefois, lorsque 2 endoprothèses sont nécessaires, il y a moyen d’améliorer les techniques existantes. La technique du *crush* a déjà connu une amélioration depuis l’introduction du *double-kissing* (*DK*) et du *minicrush*. Nous avons cherché à raffiner et à simplifier la technique du *minicrush*, tout en maintenant ses avantages et en évitant ses désavantages, par la mise au point d’une technique de *DK nanocrush*.

**Méthodes:**

La méthode du *DK nanocrush* permet de complètement couvrir une lésion de bifurcation sans couches métalliques excessives. Pour ce faire, on procède à la mise en place de l’endoprothèse dans la branche latérale (BL) avec une protrusion minimale dans la branche principale (BP), à l’implantation de l’endoprothèse dans la BL à l’aide d’un ballonnet non déployé dans la BP, à l’inflation immédiate du *kissing-balloon* avec la formation d’une néo-carina minimale, à l’implantation de l’endoprothèse dans la BP, à la traversée à nouveau la partie proximale de la BL sans traverser la couche métallique double, et on termine par le *kissing*. Nous démontrons cette technique par implantation en banc d’essai, reconstruction par microscope tomographique, modélisation numérique en mécanique des fluides (MNMF), et sur le plan clinique par l’utilisation de l’imagerie angiographique et intravasculaire.

**Résultats:**

Le *DK nanocrush* a été réalisable sur le plan pratique et a abouti à la couverture complète de l’ostium. L’analyse par MNMF a démontré un débit sanguin minimalement perturbé. La technique qui s’est avérée une réussite chez 9 patients ayant des lésions de bifurcation a mené à d’excellents résultats angiographiques et à l’absence d’événements indésirables durant 12 mois.

**Conclusions:**

En raison de ses nombreux avantages pratiques et théoriques, la technique du *DK nanocrush* peut représenter le perfectionnement ultime de la technique originale du *crush*. Il reste à éprouver cette technique par rapport à d’autres techniques de bifurcation lors d’études prospectives.

Lesions at coronary artery bifurcations are common and challenging to treat with the use of stents. The provisional approach has a strong evidence base,^1^, ^2^, ^3^ but many “true” bifurcation lesions require an up-front 2-stent strategy.^4^^,^^5^ In recent years, the culotte, T-stenting and protrusion (TAP),^6^ and improvements in the classic crush technique^7^^,^^8^ (minicrush and double-kissing [DK] crush) have translated into more assured procedures with improved clinical outcomes. Yet even the minicrush, according to a European Bifurcation Club (EBC) consensus document, may give rise to a double metal layer.^9^ There is, therefore, scope for refinement with both techniques, notably preserving lesion coverage while avoiding procedural complexity and multiple metal layers at the side branch (SB) ostium. Recently, a very minimal, or nanocrush, technique has been described, which aims to minimise protrusion of the SB stent into the main branch (MB).^10^ As a further refinement of the nanocrush and DK crush techniques, we have developed a DK nanocrush technique.

The aims of the present study were to demonstrate the deployment, geometry and hemodynamics of the DK nanocrush bifurcation stenting technique *in silico*, *in vitro*, and *in vivo*.

## Methods

### Theory

**Figure 1:**
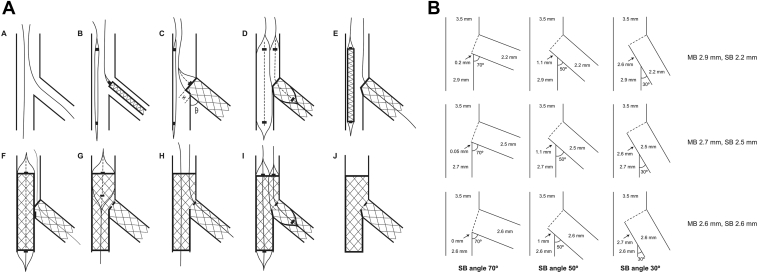
The double-kissing nanocrush technique. (**A**) The procedure. For commentary, see the *Theory* section in the *Methods*. (**B**) Geometric analysis, showing the length and position of the neocarina for 3 combinations of branch size and angle. SB, side branch.

We developed a DK nanocrush technique, suitable for almost any true coronary bifurcation requiring 2 stents. The steps are illustrated in Figure 1A, as follows. (A) Wires are passed down both MB and SB and the lesions are predilated. (B) A stent is advanced to the SB, and a balloon, slightly smaller than the distal MB, which could be the MB predilation balloon, is placed, but not inflated, in the MB across the ostium of the SB. The SB stent is withdrawn to the ostium of the SB and carefully positioned under angiography so that the proximal lateral edge of the stent is exactly at, but no farther back than, the proximal take-off of the SB. That is the first key step. (C) The SB stent is then deployed. The proximal edge of the SB stent therefore encroaches into the MB a small distance (*x*). (D) The SB balloon is pulled back half a balloon length and both SB and MB balloons are inflated in “kissing” formation. The purpose of this manoeuvre is to ensure that there is sufficient space to advance the MB stent while keeping the proximal lateral border of the SB stent well opposed at the bifurcation. Both balloons are withdrawn. (E) The MB stent, sized 1:1 to the distal MB, is then positioned and (F) implanted across the bifurcation. The SB wire may then be withdrawn. (G) Proximal optimisation with a short balloon sized 1:1 to the proximal MB may then be performed, care being taken to position the balloon proximal to the carina. (H) The SB is then rewired through the proximal part of the SB ostium, thereby avoiding the neocarina. That is the second key step. It is easy to perform, because there is only 1 layer of stent to cross and the proximal optimisation will have left an angulated entry to the SB. (I) A final kissing inflation is performed with 2 balloons sized 1:1 with their respective distal vessels. (J) The final result leaves a short neocarina, of 2 stent layers, slightly angulated between MB and SB. The procedure is compatible with the use of a 6-F guide catheter.

### Geometry

See Figure 1B: When the angle between SB and MB is θ degrees, the diameter of the proximal MB is denoted by MB_p_, the diameter of the distal MB by MB_d_, and the diameter of the SB by SB_d_, the length of the expected neocarina (*X*) may be calculated as:

$$X=SB_{d}\;\tan\left(90-\theta \right)-\frac{\left({\mathrm{MB}}_{p}-{\mathrm{MB}}_{d}\right)}{\text{sinθ}}$$

We calculated carinal length for this technique for a 3.5-mm MB_p_ leading to 3 commonly encountered combinations of distal vessel size, all obeying the law of bifurcations as set out by Huo and Kassab,^11^ and recommended by Hildick-Smith^3^ and Kassab and Finet et al.,^12^ with MB_d_/SB_d_ 2.9/2.2 mm, 2.7/2.5 mm, and 2.6/2.6 mm at angles of 30, 50, and 70 degrees.

### Benchtop demonstration

We implanted pairs of stents (Coroflex Blue; B-Braun, Melsungen, Germany) in silicone models of bifurcations on the benchtop in the same 9 configurations as outlined above (3.5/2.9/2.2 mm, 3.5/2.7/2.5 mm, and 3.5/2.6/2.6 mm each at 30, 50, and 70 degrees) under direct vision, with the use of the DK nanocrush technique, using a 3.0 mm stent in MB and a 2.5 mm stent in SB, varying the pressures to achieve 1:1 sizing with the respective downstream vessels. We documented this with photography at each deployment stage. At the end, each stented bifurcation was subjected to microscopic computed tomography (microCT; Quantum-FX; PerkinElmer, UK) and 3D image reconstruction.

### Computational fluid dynamics

Two stented silicone phantoms, namely the case with vessel size of 3.5/2.6/2.6 mm and angle of 30 degrees and the one with vessel size of 3.5/2.9/2.2 mm and angle of 70 degrees, were selected to perform pulsatile computational fluid dynamics (CFD) simulations, because they represent 2 extreme scenarios in terms of distal vessel size and bifurcation angle. A 3D reconstruction method,^13^ initially developed for nonbifurcated geometries and here adapted to bifurcations, was used to create the stented bifurcated geometries for the execution of the simulations. Detailed information about this method is reported in the Supplemental Methods. CFD was performed according to a previously described approach.^14^ A typical human left anterior descending coronary artery (LAD) flow waveform was applied at the inlet^15^ with an average flow rate of 50 mL/min.^16^ A flow-split, which was calculated with the use of the Huo-Kassab scaling law^11^ (0.5[1]:0.49 and 0.6[2]:0.38, for the first and second analysed cases, respectively), was imposed at the distal MB and SB. The walls were assumed to be rigid with a no-slip wall boundary condition. The blood was modeled as a non-newtonian fluid with constant density. For both cases, the flow patterns, regions of disturbed flow, and wall shear stress were analysed.

### Clinical feasibility and imaging

We treated 9 patients with true bifurcation disease with the use of the DK nanocrush technique. We documented acute feasibility, procedural success, and 1-year outcomes.

## Results

### Geometry

The DK nanocrush technique, with full SB coverage, resulted in a length of the neocarina, for all combinations of sizes of 2 significant branches of a 3.5-mm vessel, from 0.2 mm or nil (SB angle 70 or 90 degrees, respectively) through 1 mm (angle 50 degrees) to 2.7 mm (30 degrees; Fig. 1B), without covering the proximal part of the SB ostium with a second metal layer.

### Benchtop deployment, microCT, and computed blood flow

**Figure 2:**
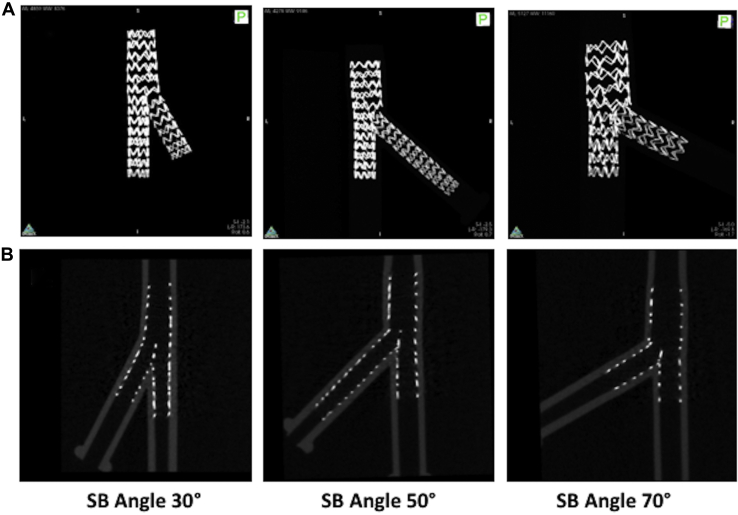
(**A**) Photographs of the key stages of the double-kissing nanocrush technique in a silicone bifurcation. (**B**) Microscopic computerized tomographic images of the deployments in silicone bifurcations deployments at 30, 50, and 70 degrees. SB, side branch.

**Figure 3:**
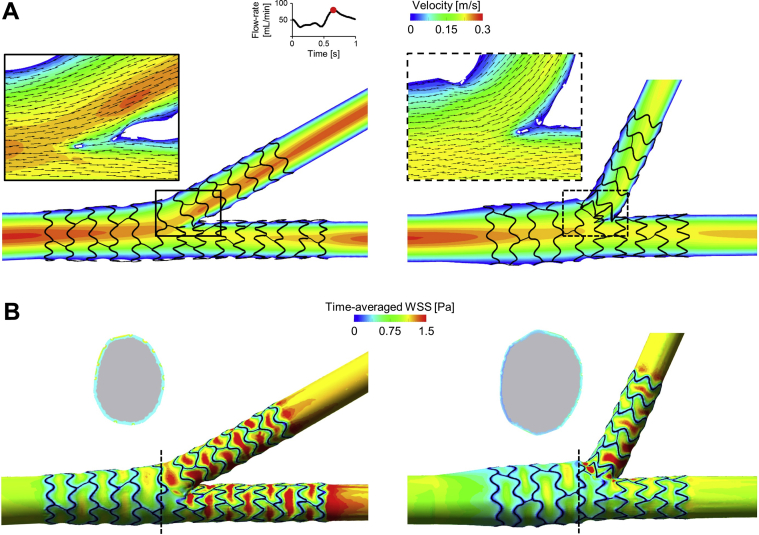
Computational fluid dynamics results of the stented silicone phantoms with vessel sizes of 3.5/2.6/2.6 mm and angle of 30 degrees (**left**), and vessel sizes of 3.5/2.9/2.2 mm and angle of 70 degrees (**right**). (**A**) Velocity contours in the middle plane at peak flow rate. The panels show a magnified image of the velocity field with in-plane velocity vectors at the bifurcation region. (**B**) Contour maps of time-averaged wall shear stress (WSS). A cross-sectional view at the proximal main branch with the lumen border coloured with the time-averaged WSS is also displayed for each case.

The DK nanocrush technique was practically feasible as described when performed under direct vision (Fig. 2A). The deployment produced a stented bifurcation as described (Fig. 2B). Figure 3A shows the velocity field at peak flow rate for the bifurcation phantom with vessel sizes of 3.5/2.6/2.6 mm and angle of 30 degrees and that with vessel sizes of 3.5/2.9/2.2 mm diameters and angle of 70 degrees. In both cases, blood flow was minimally disturbed by the stent struts without presenting any recirculation at the SB entrance. Only a small stagnation zone was observable at the neocarina of the phantom with the 70-degree bifurcation angle. As illustrated in Figure 3B, the lumen regions with low wall shear stress were located close to the stent struts in both of the investigated phantoms. The phantom with 70-degree bifurcation angle was characterized by a greater percentage lumen area exposed to time-averaged wall shear stress < 0.4 Pa compared with the case with 30 degrees (22.9% vs 15.8%, respectively). This higher value is related to the larger expansion of the proximal MB that occurred in the phantom with 70-degree bifurcation angle (Fig. 3B, cross-sectional views). The percentage area of proximal MB exposed to time-averaged wall shear stress < 0.4 Pa was 32.3% for the case with 70-degree bifurcation angle, compared with 20.1% for the phantom with 30-degree bifurcation angle. Conversely, similar values were found by limiting the analysis to the SB and distal MB stented regions (16.5% vs 12.9% for the phantoms with bifurcation angles of 70 and 30 degrees, respectively).

### Clinical deployment

**Figure 4:**
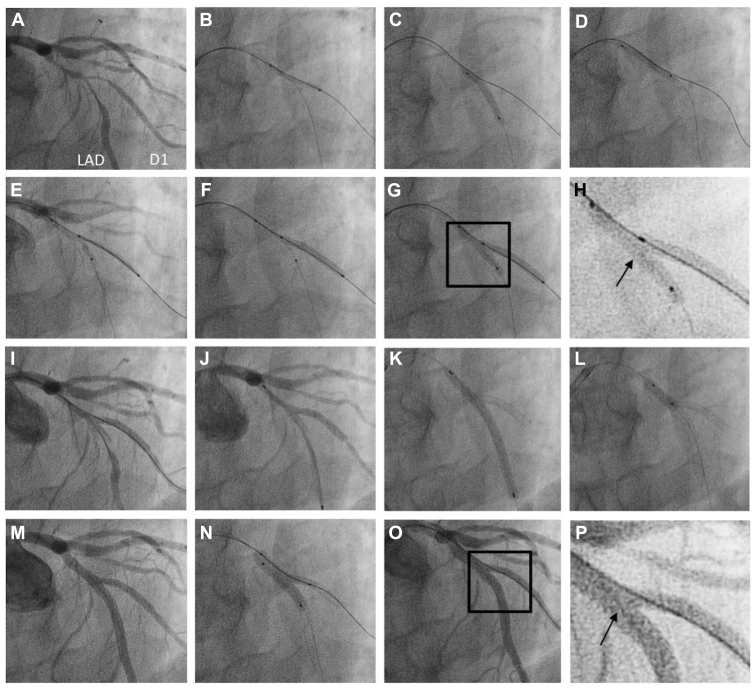
(**A**) An example of the double-kissing nanocrush technique in a 66-year-old patient with severe disease at the left anterior descending coronary artery (LAD)/diagonal (D) 1 bifurcation. (**B-D**) Both vessels were wired and then predilated. (**E**) A 2.5 × 20-mm Promus Premier (Boston Scientific) stent was positioned in D1 with a 2.5-mm balloon (the one used to predilate the LAD) in the main branch (MB). Care was taken to ensure that the proximal end of the side branch (SB) stent was precisely positioned at the take-off of the SB. (**F**) The SB stent was deployed. (**G, H**) The MB balloon was inflated, the SB balloon was pulled back, and a kissing inflation was performed. (**I**) Angiography demonstrated patency of both vessels. A 3 × 48-mm Xience (Abbott) stent was (**J**) positioned and (**K**) deployed in the MB. (**L**) Proximal optimisation was performed with a 3.5 × 12-mm balloon with (**M**) a good angiographic result. (**N**) The SB was rewired through the proximal part of the SB ostium and a final kissing inflation was performed with 3.0- and 2.5-mm balloons with (**O**) an excellent angiographic result and (**P**) a short neocarina.

**Figure 5:**
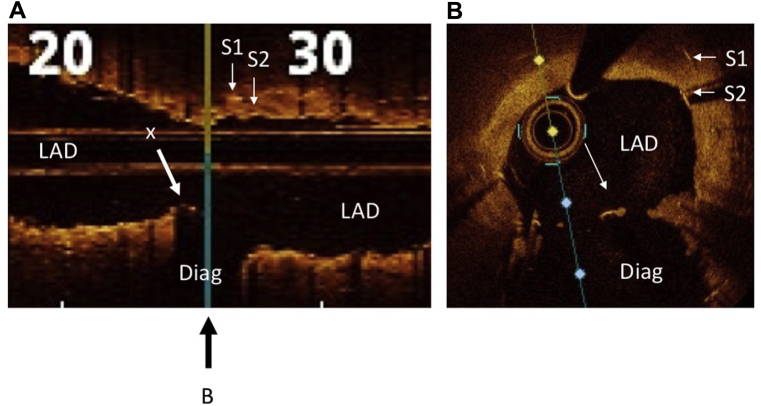
Optical coherence tomography of case 7 after double-kissing nanocrush stent deployment to the left anterior descending coronary artery (LAD) and diagonal (Diag). (**A**) Longitudinal section through the LAD, showing origin of stented diagonal branch. **X** indicates the neocarina; the **black arrow** below the figure indicates the position of cross-section shown in **B**. **S1** and **S2** are the original and new main branch stents, respectively. (**B**) Cross-section at the level of the neocarina (**X**). This example was originally a severe case of 1,1,1 bifurcation in-stent restenosis in a stent placed with the use of a provisional technique across the diagonal branch, which explains the double strut layer in the LAD.

The DK nanocrush technique was deployed in 9 patients with Medina 1,1,1 bifurcation disease. The clinical and procedural details are presented in Table 1. A single dose of periprocedural heparin was given, and all patients were prescribed dual-antiplatelet therapy in accordance with clinical presentation and European Society of Cardiology and national guidelines. The technique was clinically successful in all cases, including 1 case (patient 7) that had in-stent restenosis in a previously placed LAD stent straddling the diagonal branch. A learning curve was observed regarding wire recrossing, so final balloon kissing was not possible in 3 early cases, but a proximal optimisation technique was deployed in all. The angiographic results were excellent, Thrombolysis in Myocardial Infarction grade 3 flow was achieved in both branches, and there were no adverse outcomes at 1 year. A representative example, an LAD bifurcation lesion, is shown with the procedure captured in sequence (patient 3; Fig. 4). An example of optical coherence tomographic images at the end of the procedure (longitudinal section and cross-section, patient 7) are shown in Figure 5. Of note, in all cases, is the complete metal coverage and small unobstructive neocarina.

**Table 1:** Clinical and procedural details of patients who underwent the double-kissing nanocrush technique

Patient	Sex	Age (y)	Presentation	Lesion	Stents (mm)	Wire recross	POT	Final kiss	Angiographic success	TIMI flow	12-mo events
1	M	66	NSTE-ACS	LAD/D	3.5/2.75	N	Y	N	Y	3	N
2	M	76	STEMI	LAD/D	3.5/2.75	N	Y	N	Y	3	N
3	M	56	Stable	LAD/D	3/2.5	Y	Y	Y	Y	3	N
4	M	48	NSTE-ACS	LAD/D	3/2.5	Y	Y	Y	Y	3	N
5	F	69	Stable	LAD/D	3/2.5	Y	Y	Y	Y	3	N
6	M	66	Stable	LAD/D	2.75/2.5	Y	Y	N	Y	3	N
7	M	59	Stable	LMS/Cx	4.5/3	Y	Y	Y	Y	3	N
8	F	50	NSTE-ACS	LMS/Cx	4/3	Y	Y	Y	Y	3	N
9	M	58	Stable	LAD/D	3.5/2.5	Y	Y	Y	Y	3	N

Cx, circumflex; D, diagonal; LAD, left anterior descending coronary artery; LMS, left main stem; NSTE-ACS, non–ST-segment-elevation acute coronary syndrome; POT, proximal optimisation technique; TIMI, Thrombolysis in Myocardial Infarction.

## Discussion

In this study, we have demonstrated the theory and practical feasibility of a DK nanocrush technique to treat true bifurcation coronary artery disease. This technique is a refinement of minicrush and DK crush and provides full lesion coverage, minimal-difficulty rewiring, a short neocarina, minimal protrusion into the MB, and favourable hemodynamics.

This technique, which builds on the most promising recent developments in bifurcation percutaneous coronary intervention strategy, was pursued because of the continuing need for a simple effective 2-stent strategy to treat bifurcations. It appeared from guidelines that even the “minicrush” may leave a double metal layer over the whole SB ostium. We showed, in contrast, that this is not required or, indeed, possible with accurate representation of the distal branch sizes according to the rules of Huo and Kassab,^11^ and as recommended by Kassab and Finet,^12^ and meticulous SB stent positioning in the nanocrush position. When the best physiologic rules are followed (portrayed to exact scale in our figures), the only double layer forms a short neocarina varying in length from nil (SB angle 70 degrees) to 2.7 mm (SB angle 30 degrees [an unusually extreme case]), in a typical bifurcation of a 3.5-mm vessel, whatever the relative calibres of the distal MB and SB. Even large neocarinas are promptly covered, so this ultrashort new carina should not be of any concern.^17^ The size of the neocarina is identical to that of the TAP, which is associated with excellent long-term outcomes.^18^ Also, avoidance of a double layer over the proximal SB orifice allows easy wire recrossing after proximal optimisation and a high success rate of final kissing with minimal protrusion into the MB.

TAP has become a popular strategy because it produces a good final result in most cases, comprises relatively few uncomplicated steps, and is feasible with the use of a 6-F guiding catheter. However, TAP is a provisional technique (A in the EBC MADS classification): The MB is treated first and the SB is rescued, post hoc, if needed. This has a strong evidence base and is appropriate for many cases. However, there are some cases which require the SB to be secured before the MB is treated. Current up-front 2-stent techniques include crush (DK and mini), culotte, simultaneous kissing stents, and V. These techniques are either more complicated or beset with potential limitations. This is where the DK nanocrush is effective, because it is appropriate in cases where securing the SB is a priority (S in the MADS classification) but with key advantages of TAP. Key procedural, technical, and clinical characteristic of bifurcation stent techniques are compared in Table 2.

**Table 2:** Comparing DK nanocrush with other 2-stent bifurcation strategies

Factor	DK nanocrush	DK/mini-crush	T	TAP	Culotte	V	SKS
Steps∗	8	10, 13 if DK	8	8	9	4	5
MB rewire	N	N	N	N	Y	N	N
SB rewire	Y (through >1 layers)	Y	Y	Y	Y	N	N
Provisional approach possible	N	N	Y	Y	Y	Simultaneous	Simultaneous
Ideal angle	Any	Narrow	Wide	Any	Narrow	Any	Any
Suitable for MB/SB size mismatch	Y	Y	Y	Y	N	Y	Y
6 Fr suitable	Y	N (7Fr)	Y	Y	Y	N (7Fr)	N (7Fr)
Potential drawbacks/other considerations	Careful SB stent positioning required	Multiple crushed stent layers. Difficulties rewiring and delivering balloons to SB	Gap at ostium	Careful SB stent positioning required	Multiple re-wiring. Double layer of stent struts proximally	Uncovered proximal disease	Large neocarina. Difficult reintervention

DK, double-kissing; MB, main branch; N, no; SB, side branch; SKS, simultaneous kissing stents; TAP, T and protrusion; Y, yes.

∗ Including initial wiring, predilatation, and proximal optimisation steps.

In contrast, DK nanocrush is an up-front 2-stent bifurcation strategy. It is well suited to cases where the priority is to secure the SB. There is no need to lose access to the MB at any point, and SB rewiring is improved by needing to cross only 1 layer of stent and facilitated by the first kiss. The key technical challenge is positioning the SB stent before deployment. Careful angiography and optimal visualisation of the bifurcation is required so that the SB stent is positioned at the ostium to ensure full proximal ostial coverage with minimal MB protrusion. Similarly to other bifurcation stent strategies, it is vital that the proximal optimisation technique step is also performed adequately to high pressure, sized 1:1 to the proximal vessel, covering the proximal portion of the SB ostium.

Good 30-day and 1-year clinical results were achieved with this technique in an unselected series of 9 “all-comer” patients with true bifurcation disease, although the final kissing SB balloon could not be advanced in 1 case with heavy calcification. Before wide-scale adoption, it will be important to ascertain the long-term outcomes in a large adequately-powered cohort and to generate prospectively collected randomised comparative data vs other 2-stent techniques.

## Conclusion

The DK nanocrush technique may offer all the advantages of minicrush and DK crush without the disadvantages. It is reasonably easy and quick to perform, and has the advantages of full lesion coverage, easier SB recross, and minimised carinal protrusion and double metal layers. As with all 2-stent techniques, heavily calcified lesions require comprehensive preparation. Long-term results are awaited.
