# Supplementary material for: Double-Kissing Nanocrush for Bifurcation Lesions: Development, Bioengineering, Fluid Dynamics, and Initial Clinical Testing
Source: Can J Cardiol. 2020 Jun;36(6):852–9. doi: 10.1016/j.cjca.2019.08.037 (PMC7308801; doi:10.1016/j.cjca.2019.08.037)
Supplement: Supplemental Material [file mmc1.pdf]

Supplementary data

Supplemental methods

Computational fluid dynamics: 3D reconstruction of the stented phantoms

The stented silicone phantoms with vessel size of 3.5/2.6/2.6 mm and angle of 30 degrees, and vessel size of 3.5/2.9/2.2 mm and angle of 70 degrees were chosen to perform pulsatile computational fluid dynamics simulations. Since the resolution of the micro CT scans of the stented phantoms (i.e. 40 microns) was not sufficient for a direct 3D reconstruction of both the stents and the lumen, the following approach was adopted to generate the fluid domains for the numerical simulations.

To reconstruct the stents, the micro CT data were segmented using the commercial software Mimics (Materialise, Leuven, Belgium). A first 'rough' 3D reconstruction of the stents was obtained (Figure S1-A) and then used to extract the stent centreline (i.e. stent skeleton) (Figure S1-B). Finally, the 3D model of the stent was generated by connecting cross-sectional profiles of the stent struts placed along the skeleton lines (Figure S1-C, D). This last step was performed by running an in-house semi-automatic algorithm developed in the graphical editor Grasshopper (www.grasshopper3d.com) within the computer-aided design software Rhinoceros (Robert McNeel & Associates, Seattle, WA, USA), as previously done for the 3D reconstruction of stents from optical coherence tomography images (1,2).

To generate the lumen model, a quasi-static finite element analysis was performed using the finite element solver ABAQUS/Explicit (Dassault Systemes Simulia Corp., Providence, RI, USA). The method initially developed for non-bifurcated geometries (3) was adapted to bifurcations. Specifically, a bifurcated vessel model with initial diameter in the stented region slightly smaller than the stents (Figure S2-A) was expanded by applying an internal

pressure until it reached a diameter larger than the stents. Subsequently, the vessel geometry retracted due to elastic recoil and made contact with the stents (Figure S2-B), which were modelled as rigid structures.

The vessel geometry was designed by considering that the only aim of the finite element model was the generation of the fluid domain. Hence, the stresses and strains within the arterial wall were not analysed. The undeformed geometry of the vessel was discretized with ~ 11,000 four-node membrane elements with full integration using HyperMesh (Altair Engineering, Troy, MI, USA). The material behaviour was described using an isotropic linear elastic constative law with Young's modulus of 3 MPa and Poisson coefficient of 0.45 (4). The material density was set to 1.12E-06 kg/m^3 (5). A constant thickness of 0.15 mm (6) was assigned to the membrane elements. The interaction between the artery and the stent was defined as a general contact with 'hard' normal behaviour and tangential behaviour with static friction coefficient of 0.2(5).

Supplemental Figures

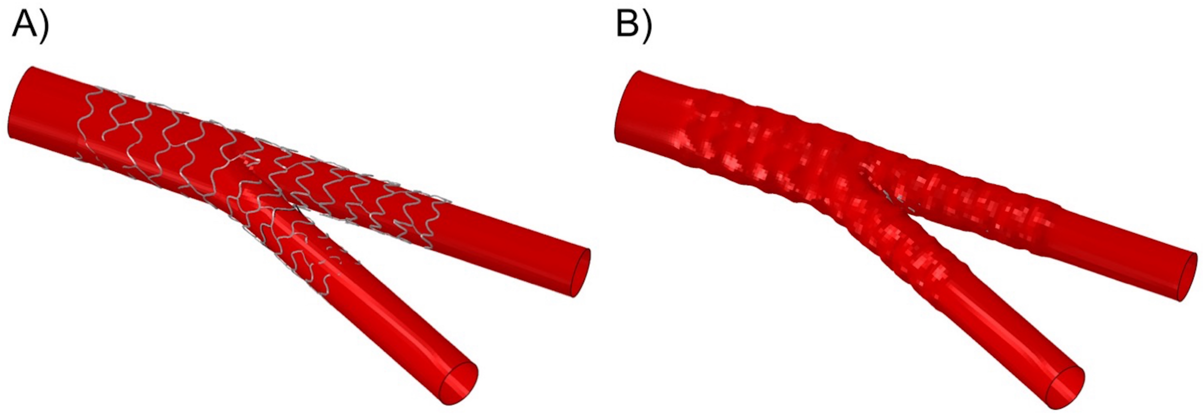

Figure S1: Finite element analysis for obtaining the bifurcated lumen geometry for subsequent computational fluid dynamics simulation: A) unexpanded vessel configuration; B) final vessel configuration after the expansion and recoil steps.

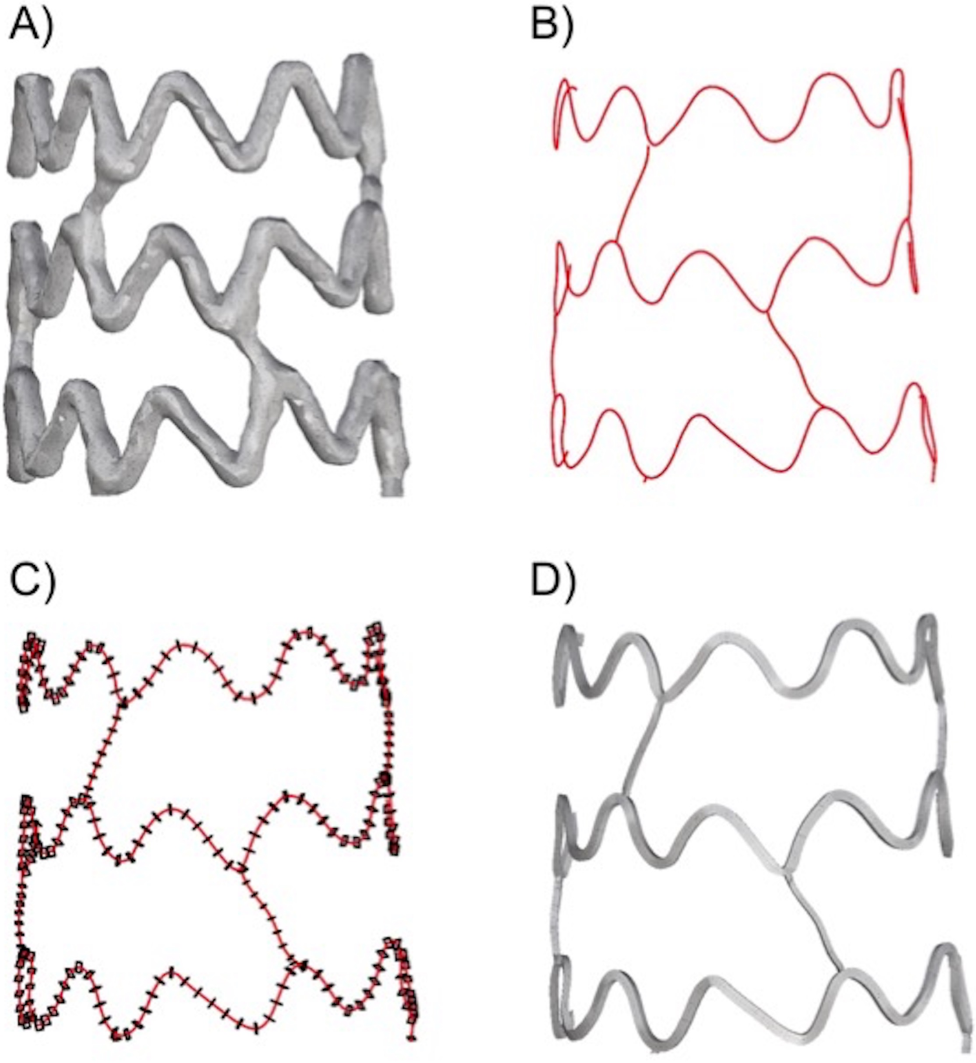

Figure S2: Procedure followed for reconstructing the stents. A) 'Rough' 3D reconstruction obtained using Mimics; while the 3D stent shape is preserved, the stent strut size and cross-sectional shape us not well-captured. B) Extraction of the stent skeleton. C.) Cross-sectional profiles of the stent struts places along the skeleton lines. D) Final 3D stent reconstruction.

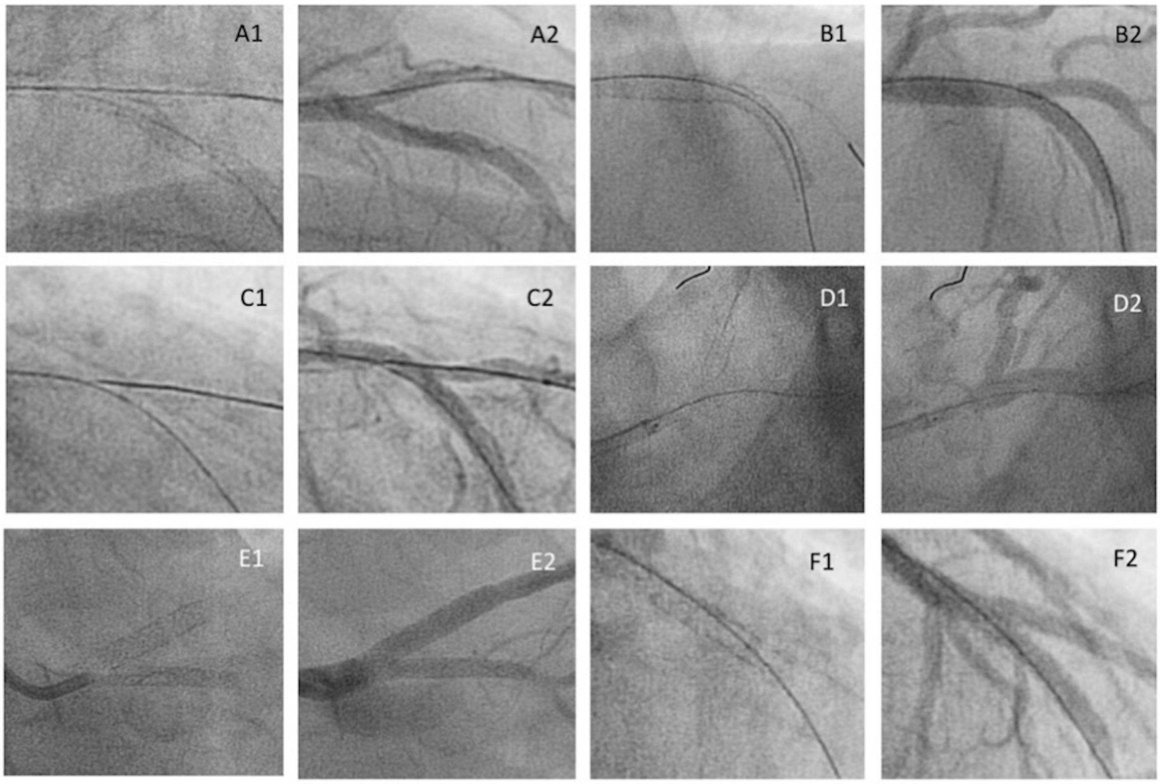

Figure S3: Final angiographic results, with (left) and without (right) contrast, for patients 2-7. For Patient 1, see Figure 4. A) Patient 2 (LAD/D1). B) Patient 3 (LAD/D1). C) Patient 4 (LAD/D1). This was a patient with heavily calcified lesion which required extensive lesion preparation with scoring balloons. After MB stenting a wire was passed to the SB, but a balloon could not be advanced, so there was no final kiss. D) Patient 5 (LMS/Cx). E) Patient 6 (LMS/Cx). F) Patient 7 (LAD/D1). This was a patient with in-stent restenosis of a prior stent in the LAD across the diagonal.

Reference

1. Migliori S, Chiastra C, Bologna M et al. A framework for computational fluid dynamic analyses of patient-specific stented coronary arteries from optical coherence tomography images. Med Eng Phys 2017;47:105-116.
2. Migliori SR, R.; Bolonga, M.; Montin, E.; Burzotta, F.; Hildick-Smith, D.; Dubini, G.; Mainardi, L.; Migliavacca, F.; Cockburn, J.; Chiastra, C. A Patient-Specific Study Investigating the Relation between Coronary Haemodynamics and Neo-Intimal Thickening after Bifurcation Stenting with a Polymeric Bioresorbable Scaffold. Applied Sciences 2018;8:1510.
3. Morlacchi S, Keller B, Arcangeli P et al. Hemodynamics and in-stent restenosis: micro-CT images, histology, and computer simulations. Ann Biomed Eng 2011;39:2615-26.
4. Karimi A, Rahmati SM, Sera T, Kudo S, Navidbakhsh M. A combination of experimental and numerical methods to investigate the role of strain rate on the mechanical properties and collagen fiber orientations of the healthy and atherosclerotic human coronary arteries. Bioengineered 2017;8:154-170.
5. Chiastra C, Grundeken MJ, Collet C et al. Biomechanical Impact of Wrong Positioning of a Dedicated Stent for Coronary Bifurcations: A Virtual Bench Testing Study. Cardiovasc Eng Technol 2018;9:415-426.
6. Iannaccone F, Chiastra C, Karanasos A et al. Impact of plaque type and side branch geometry on side branch compromise after provisional stent implantation: a simulation study. EuroIntervention 2017;13:e236-e245.
